# Relationship of Admission Serum Anion Gap and Prognosis of Critically Ill Patients: A Large Multicenter Cohort Study

**DOI:** 10.1155/2022/5926049

**Published:** 2022-12-14

**Authors:** Ruohan Li, Xuting Jin, Jiajia Ren, Guorong Deng, Jiamei Li, Ya Gao, Jingjing Zhang, Linyun Du, Jueheng Liu, Xiu Liu, Xiaochuang Wang, Gang Wang

**Affiliations:** Department of Critical Care Medicine, The Second Affiliated Hospital of Xi'an Jiaotong University, Xi'an, China

## Abstract

**Background:**

There were controversies over the relationship between Anion gap (AG) and mortality in critically ill patients. Therefore, a large multicenter cohort study was conducted to evaluate the association of AG and mortality in large-scale intensive care units (ICUs) patients.

**Methods:**

This retrospective cohort study included adult ICU patients enrolled from eICU Collaborative Research Database. According to initial serum AG upon ICU admission, patients were divided into three groups: AG < 8 mmol/L, 8 ≤ AG ≤ 16 mmol/L, and AG > 16 mmol/L. Logistic regression models were built to investigate the association between serum AG and ICU and hospital mortalities. Serum AG was added into Acute Physiology and Chronic Health Evaluation (APACHE) IV score and the model discrimination was assessed by the area under the curve (AUC) of receiver operating characteristic curves. The relationship between serum AG and mortalities in patients with different acid-base status and serum lactate were also evaluated. An external validation was performed with the Critical care database comprising patients with infection at Zigong Fourth People's Hospital.

**Results:**

A total of 8520 patients entered the final cohort. There are 42 patients with serum AG < 8 mmol/L, 3238 patients with 8 ≤ AG ≤ 16 mmol/L, and 5240 patients with AG > 16 mmol/L. Serum AG > 16 mmol/L is related with increased ICU mortality (odds ratio [OR], 1.530; 95% confidence interval [CI], 1.305–1.794) and hospital mortality (OR, 1.618; 95% CI, 1.415–1.849), compared with 8 ≤ AG ≤ 16 mmol/L. Adding Serum AG to APACHE IV score could statistically improve the prediction of ICU (0.770 [0.761–0.779] to 0.774 [0.765–0.783], *P* = 0.001) and hospital mortalities (0.756 [0.747–0.765] to 0.761 [0.751–0.770], *P* = 0.012). The associations between serum AG and mortalities remain robust in patients with different acid-base statuses and serum lactate. The findings are validated in the external cohort.

**Conclusions:**

Initial serum AG > 16 mmol/L after ICU admission is associated with increased mortality in critically ill patients.

## 1. Introduction

Critically ill patients were more likely to develop acidosis, due to their underlying illness [1]. For example, hypoperfusion or hypoxia (such as shock, sepsis and severe anemia) and metabolic disorders (such as liver diseases, diabetes mellitus, and poisoning) contribute to overproduction of acid, while renal failure leads to decreased excretion of acid [2, 3]. The serum anion gap (AG), presenting the difference between unmeasured anions and unmeasured cations, is mainly composed of serum albumin, phosphate, sulfate, and organic acids [4]. In clinical, AG is commonly used to detect and analyze acid-base disorders, primarily metabolic acidosis [2, 3, 5]. After reviewing 19 documented studies, a meta-analysis stated that AG was not recommended to evaluate the risk for mortality in critically ill patients [6]. Whereas, recent studies demonstrated that increased AG was associated with high mortality in ICU patients with aortic aneurysm [7], sepsis [8], disseminated intravascular coagulation [9], and congestive heart failure [10]. Besides, a study conducted in COVID-19 patients showed that AG was one of the most decisive features for mortality prediction in a machine learning model [11]. The inconsistent conclusions might be attributed to the heterogeneities in study designs and inclusion criteria. In addition, the relationship between AG and the prognosis of critically ill patients with or without various acid-base disorders or not still remains unclear. Therefore, we aimed to conduct a large, multicenter, retrospective study in a broad ICU population using eICU Collaborative Research Database (eICU-CRD, version 2.0) to further investigate the relationship between AG and mortality. We also explored the association between AG and mortality in ICU patients with different acid-base statuses.

## 2. Methods

### 2.1. Study Design and Data Extraction

This cohort study was conducted with eICU-CRD version 2.0, which is a freely available database maintained by the Laboratory for Computational Physiology at the Massachusetts Institute of Technology (MIT; Cambridge, MA, USA). The eICU-CRD was comprised of deidentified and high granularity data for 200859 ICU admissions between 2014 and 2015 for 139367 patients in 335 distinct ICUs across the United States, which included demographic characteristics, treatment information, severity of illness measures, diseases diagnosed via International Classification of Diseases, Ninth Edition (ICD-9), laboratory tests information, and more [12]. The reidentification risk of eICU-CRD meets safe harbor standards by Privacert (Cambridge, MA) (HIPAA Certification no. 1031219-2). The requirement for individual patient consent was waived because the project did not impact clinical care and all protected health information was deidentified. All authors in this study received permission to access eICU-CRD.

We extracted the following information from eICU-CRD for each patient: age, gender, ethnicity, the first Acute Physiology and Chronic Health Evaluation (APACHE) IV, diseases diagnosed with ICD-9 during the first 24 h ICU stay (including heart failure, respiratory failure, renal failure, liver diseases, coagulopathy, sepsis, trauma, shock, hypertension, and diabetes mellitus), initial serum sodium (Na^+^), potassium (K^+^), chloride (Cl^−^), bicarbonate (HCO_3_^−^), albumin, and lactate, initial blood pH and PaCO_2_ from arterial blood gas in the first 24 h after ICU admission, treatments in the 24 hours prior to the measurement of serum electrolytes, and the status of hospital discharge. Data extraction was performed using SAS version 9.4 (SAS institute, Cary, NC, USA).

### 2.2. Patients

**Figure 1:**
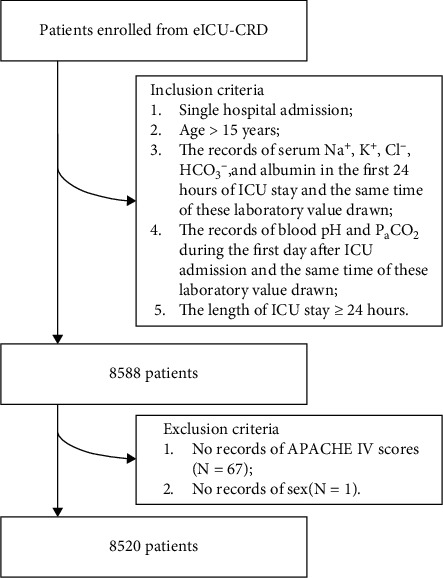
Flow diagram for patient recruitment. APACHE, Acute physiology and chronic health evaluation; eICU-CRD, eICU collaborative research database; ICU, intensive care unit.

Patients were enrolled from eICU-CRD database. The information of the first ICU admission were used for patients who had multiple ICU admission records. The first value was used when patients had multiple records of laboratory test. Inclusion criteria were as follows: patients who had (1) single hospital admission; (2) age > 15 years; (3) the records of serum Na^+^, K^+^, Cl^−^, HCO_3_^−^, and albumin in the first 24 hours of ICU stay and the same time of these laboratory value drawn; (4) the records of blood pH and P_a_CO_2_ during the first day after ICU admission and the same time of these laboratory value drawn; and (5) the length of ICU stay ≥ 24 hours. Exclusion criteria were as follows: patients who had (1) no records of APACHE IV scores, or (2) no records of gender (Figure 1).

### 2.3. Initial AG, Normal Acid-Base Status, and Outcomes

Initial serum AG was calculated as follows: AG = ([Na^+^] + [K^+^]) − ([Cl^−^] + [HCO_3_^−^]). To avoid the effect of abnormal albumin concentration on serum AG, the corrected serum AG was calculated as AG + 2.5[4.0 − albumin (g/dL)] when serum albumin was not within the range of 3.5-5.5 g/dL [13]. Patients were divided into three groups based on initial serum AG: AG < 8 mmol/L, 8 ≤ AG ≤ 16 mmol/L, and AG > 16 mmol/L [14]. According to previous studies, the normal acid-base status was defined as blood pH within the range of 7.35-7.45, HCO_3_^−^ within the range of 22-26 mmol/L, and PaCO_2_ within the range of 35-45 mmHg [15, 16]. The outcomes were ICU mortality and hospital mortality.

### 2.4. Statistical Analysis

Data for continuous variables and categorical variables were expressed as median with interquartile range and frequency with percentage, respectively. Differences were evaluated using Mann–Whitney test for continuous variables and chi-squared test or Fisher's exact test for categorical variables. Univariable and multivariable logistic regression models were used to investigate the association between serum AG and mortalities. The covariates included age (≤65 years vs. >65 years), sex (male vs. female), ethnicity (Caucasian vs. other), APACHE IV (≤73 vs. >73), heart failure, respiratory failure, renal failure, liver diseases, coagulopathy, sepsis, trauma, shock, hypertension, diabetes mellitus, serum lactate (<2 mmol/L vs. ≥2 mmol/L, the missing values of serum lactate were imputed using multiple imputation method [17]), and treatments (renal replacement therapy and mechanical ventilation). Serum AG was added into APACHE IV score, and the predictive accuracy was assessed by calculating the area under the receiver operating characteristic curves (AUROC).

Furthermore, the relationship between serum AG and mortalities in patients with different acid-base statuses were also evaluated. Spearman's Rank Sum Correlation Test was conducted to assess the correlation between serum AG (as continuous variable) and serum lactate, and the percentage of patients with serum lactate ≥ 2 mmol/L were evaluated in patients with different serum AG. Subgroup analysis were also performed to investigate the relationship between serum AG and mortalities in patients with different serum lactate (<2 mmol/L and ≥ 2 mmol/L), age (≤65 years vs. >65 years), sex (male vs. female), ethnicity (Caucasian vs. other), APACHE IV (≤73 vs. >73), heart failure (Yes vs. No), respiratory failure (Yes vs. No), renal failure (Yes vs. No), liver diseases (Yes vs. No), coagulopathy (Yes vs. No), sepsis (Yes vs. No), trauma (Yes vs. No), shock (Yes vs. No), hypertension (Yes vs. No), diabetes mellitus (Yes vs. No), renal replacement therapy (Received vs. Nonreceived), and mechanical ventilation (Received vs. Nonreceived). All statistical analyses were performed using SPSS version 22.0 (SPSS, Chicago, IL, USA).

A correlation matrix between AG and other serum biomarkers, including sodium, potassium, calcium, albumin, bicarbonate, creatinine, prolactin, prealbumin, transferrin, triglycerides, troponin I, troponin T, amylase, vitamin B12, direct bilirubin, fibrinogen, folate, alkaline phosphatase, free T4, ammonia, haptoglobin, sketones, magnesium, myoglobin, lipase, total bilirubin, total cholesterol, and total protein, blood glucose, and urea nitrogen) was determined by the Spearman's correlation coefficient. A two-sided *P* < 0.05 was considered to be statistically significant.

### 2.5. External Validation

External validation was performed using the Critical care database comprising patients with infection at Zigong Fourth People's Hospital version 1.1. This database includes 2790 ICU patients with infection at Zigong Fourth People's Hospital, Sichuan, China from January 2019 to December 2020. The establishment of this database was approved by the ethics committee of Zigong Fourth People's Hospital (Approval Number: 2020-065) [18]. Due to a retrospective design of the study, the requirement for individual patient consent was waived. All authors received permission to access the database.

All information for each patient were extracted from the database using SAS version 9.4 (SAS institute, Cary, NC, USA). Adult patients (age > 18 years) who had single hospital admission, completed records of AG and albumin in the first 24 hours of ICU stay, and the length of ICU stay ≥ 24 hours were included.

## 3. Results

### 3.1. Characteristics of Study Subjects

There were 8588 patients meeting our inclusion criteria. Then, 67 patients without the records of APACHE IV scores, and 1 patient without the records of sex were excluded. Ultimately, a total of 8520 patients entered the final cohort (Figure 1). Among all the included patients, there are 4063 (47.7%) patients with age > 65 years, 4671 (54.8%) males, and 6680 (78.4%) Caucasians. The median of APACHE IV scores is 73 (55-94). The median of initial serum AG and lactate is 17.3 (14.5-20.5) mmol/L and 2.0 (1.2-3.5) mmol/L, respectively. There are 42 patients with serum AG < 8 mmol/L, 3238 patients with 8 ≤ AG ≤ 16 mmol/L, and 5240 patients with AG > 16 mmol/L. Factors that significantly differed among these groups include age > 65 years (*P* < 0.001), APACHE IV scores (*P* < 0.001), serum lactate (*P* < 0.001), the prevalence of respiratory failure (*P* = 0.011), renal failure (*P* < 0.001), liver diseases (*P* < 0.001), coagulopathy (*P* = 0.006), sepsis (*P* < 0.001), shock (*P* < 0.001), trauma (*P* < 0.001), hypertension (*P* < 0.001), mechanical ventilation (*P* < 0.001), and renal replacement therapy (*P* < 0.001). The details of characteristics of this study subjects are showed in Table 1.

### 3.2. The Association between Initial Serum AG and Mortalities in All Patients

Among all patients, ICU mortality is 16.7% in patients with serum AG < 8 mmol/L, 8.1% in those with 8 ≤ AG ≤ 16 mmol/L, and 18.2% in those with AG > 16 mmol/L (*P* < 0.001). Hospital mortality is 19.0% in the group of serum AG < 8 mmol/L, 12.5% in those of 8 ≤ AG ≤ 16 mmol/L, and 26.6% in those of AG > 16 mmol/L (*P* < 0.001, Table 1). In unadjusted logistic regression models, serum AG < 8 mmol/L is associated with increased ICU mortality (odds ratio [OR], 2.281; 95% confidence interval [CI], 1.003-5.186), and serum AG > 16 mmol/L is related with higher ICU mortality (OR, 2.539; 95% CI, 2.197-2.934) and hospital mortality (OR, 2.531; 95% CI, 2.243-2.855) risks than those with 8 ≤ AG ≤ 16 mmol/L. In multivariable models, patients with serum AG < 8 mmol/L also have a higher ICU mortality (OR, 2.504; 95% CI, 1.039-6.035), and patients with serum AG > 16 mmol/L also have increased ICU mortality (OR, 1.530; 95% CI, 1.305-1.794) and hospital mortality (OR, 1.618; 95% CI, 1.415-1.849), compared with those with 8 ≤ AG ≤ 16 mmol/L (Table 2).

### 3.3. Serum AG Improved the AUROC of the APACHE IV Score for Predicting ICU and Hospital Mortalities

We compared the AUROC values between APACHE IV score and APACHE IV score plus AG for predicting ICU and hospital mortalities. The results show that the combination between serum AG and APACHE IV score could significantly improve the prediction of ICU mortality (0.774 [0.765–0.783] vs. 0.770 [0.761–0.779], *P* = 0.001) and hospital mortality (0.761 [0.751–0.770] vs. 0.756 [0.747–0.765], *P* = 0.012) than APACHE IV score alone.

### 3.4. The Relationship between Initial Serum AG and Mortalities in Patients with Different Acid-Base Status

**Figure 2:**
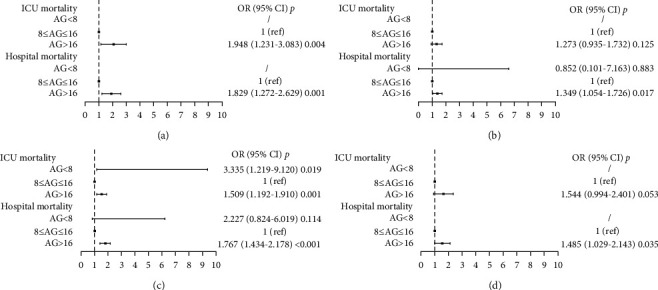
The association between serum AG and mortalities in patients with different acid-base status after adjustment. (a) Patients with normal acid-base status; (b) patients who had acid-base disorder with a normal blood pH; (c) patients with blood pH < 7.35; (d) patients with blood pH > 7.45. The adjusted covariates including age, sex, ethnicity, APACHE IV score, heart failure, respiratory failure, renal failure, liver diseases, coagulopathy, sepsis, trauma, shock, hypertension, diabetes mellitus, and serum lactate. AG, anion gap; CI, confidence interval; OR, odds ratio.

In most cases, serum AG is usually ignored when acid-base status is normal. Thus, to further investigate the relationship between serum AG and mortalities in patients with different acid-base statuses, especially in those with a normal acid-base status, patients were further divided into four subgroups: normal acid-base status (*n* = 1167), acid-base disorder with a normal blood pH (*n* = 2432), blood pH < 7.35 (*n* = 3785), and blood pH > 7.45 (*n* = 1136). The differences of ICU mortality and hospital mortality between patients with 8 ≤ AG ≤ 16 mmol/L and AG > 16 mmol/L in these four subgroups are statistically significant, respectively (all *P* < 0.05, Table 3).  Figure 2(a) show that patients who had a serum AG > 16 mmol/L had an elevated ICU mortality (OR, 1.948; 95% CI, 1.231-3.083) and hospital mortality (OR, 1.829; 95% CI, 1.272-2.629) than those with 8 ≤ AG ≤ 16 mmol/L after adjusting for multiple covariates in the subgroup of normal acid-base status. In subgroup of acid-base disorder with a normal blood pH, serum AG > 16 mmol/L is related with increased hospital mortality (OR, 1.349; 95% CI, 1.054-1.726; Figure 2(b)). The relationship between serum AG and ICU mortality (OR, 1.509; 95% CI, 1.192-1.910) and hospital mortality (OR, 1.767; 95% CI, 1.434-2.178) still have statistical significance in the subgroup of blood pH< 7.35 (Figure 2(c)). The association of serum AG > 16 mmol/L with increased hospital mortality (OR, 1.485; 95% CI, 1.029-2.143) also exist in the subgroup of blood pH > 7.45 (Figure 2(d)).

### 3.5. The Correlation of Serum AG with Lactate

**Figure 3:**
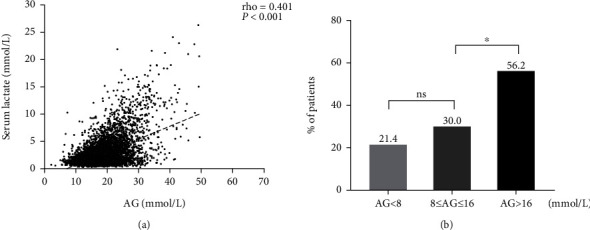
The correlation between serum AG and serum lactate. (a) Spearman's Rank Sum Correlation Test was used to assess the relationship between serum AG and serum lactate. Rho is the Spearman's correlation coefficient; (b) the different percentage of serum lactate≥2 mmol/L in patients with different serum AG. ^∗^*P* < 0.001; ns, no statistical significance. AG, anion gap.

Previous studies demonstrated a contradictory conclusion about the correlation of serum AG with lactate [19, 20]. Thus, our study conducted Spearman's Rank Sum Correlation Test to assess the correlation between serum AG (as continuous variable) and serum lactate. We find that there was a weak correlation between initial serum AG and serum lactate (Spearman's rho = 0.401, *P* < 0.001, Figure 3(a)). And serum lactate is more than 2 mmol/L in 57.9% of patients with serum AG > 16 mmol/L (Figure 3(b)).

The association between serum AG and mortalities are conducted in patients stratified by serum lactate. Among patients with serum lactate ≥ 2 mmol/L, serum AG > 16 mmol/L is associated with higher ICU mortality (OR, 2.382; 95% CI, 1.936-2.930) and hospital mortality (OR, 2.393; 95% CI, 2.000-2.863) than 8 ≤ AG ≤ 16 mmol/L. After covariates adjusted, the association between serum AG and ICU mortality (AG > 16 mmol/L vs. 8 ≤ AG ≤ 16 mmol/L: OR, 1.902; 95% CI, 1.528-2.367) and hospital mortality (AG > 16 mmol/L vs. 8 ≤ AG ≤ 16 mmol/L: OR, 1.870; 95% CI, 1.544-2.265) remain robust. In patients with serum lactate < 2 mmol/L, unadjusted models showed that high serum AG is related with increased ICU mortality (AG> 16 mmol/L vs. 8 ≤ AG ≤ 16 mmol/L: OR, 1.492; 95% CI, 1.193-1.867) and hospital mortality (AG > 16 mmol/L vs. 8 ≤ AG ≤ 16 mmol/L: OR, 1.701; 95% CI, 1.425-2.029). Multivariable models show that serum AG > 16 mmol/L is still related with high hospital mortality (OR, 1.395; 95% CI, 1.153-1.686) than 8 ≤ AG ≤ 16 mmol/L (Table 4).

### 3.6. Subgroup Analyses

To further address the potential influence of different covariates on the results, subgroup analyses were conducted in patients classified by age, sex, ethnicity, APACHE IV, heart failure, respiratory failure, renal failure, liver diseases, coagulopathy, sepsis, trauma, shock, hypertension, and diabetes mellitus. The results show that the association between serum AG and mortalities are consistent in patients varied by the above covariates, except for those with coagulopathy, trauma, hypertension, diabetes mellitus, mechanical ventilation, and renal replacement therapy (Supplementary Table 1).

### 3.7. Correlation Matrix

The results show that the correlations between AG and other serum biomarkers (sodium, potassium, calcium, albumin, bicarbonate, creatinine, prolactin, prealbumin, transferrin, triglycerides, troponin I, troponin T, amylase, vitamin B12, direct bilirubin, fibrinogen, folate, alkaline phosphatase, free T4, ammonia, haptoglobin, sketones, magnesium, myoglobin, lipase, total bilirubin, total cholesterol, and total protein, blood glucose, and urea nitrogen) are weak (Supplementary Figure 1).

### 3.8. External Validation

A total of 1873 patients were included in the external validation cohort. Patients' characteristics are shown in Supplementary Table 2. In the external validation cohort, higher serum AG is also associated with increased hospital mortality (OR, 1.667; 95% CI, 1.065-2.607) after adjusting for age, gender, heart failure, respiratory failure, renal failure, liver diseases, coagulopathy, sepsis, trauma, shock, hypertension, and diabetes mellitus (Table 5).

## 4. Discussion

In this large, multicenter, retrospective cohort study, we find that a large proportion of ICU patients (61.5%) have an elevated initial AG (serum AG > 16 mmol/L). Initial serum AG > 16 mmol/L during the first 24 hour after ICU admission is independently associated with higher ICU mortality and hospital mortality in critically ill patients, and this relationship still exists in patients with different acid-base statuses.

Previous studies reported inconsistent results regarding the association of serum AG with mortalities in critically ill patients. A single center prospective study with 175 ICU patients showed that there was no difference between survivors and nonsurvivors in AG (*P* = 0.619) or corrected AG (*P* = 0.106) on ICU admission [21]. In contrast, Cusack et al. [22] performed a single center prospective study in 100 medical and surgical ICU patients, and demonstrated that AG (*P* = 0.007) and AG corrected with albumin (*P* = 0.007) on ICU admission were higher in survivors than in nonsurvivors. Furthermore, Masevicius et al. [1] conducted a prospective cohort study and discovered that increased unmeasured anions (calculated as AG − lactate) was positively correlated with the risk of mortality in 4901 medical and surgical ICU patients (OR, 1.04; 95% CI, 1.02-1.06). Several recent retrospective studies also demonstrated that elevated AG within the first day of ICU admission was related with increased mortality among critically ill patients with sepsis [8], congestive heart failure [10], aortic aneurysm [7], cardiogenic shock [23], and acute pancreatitis [24]. The different study designs and the heterogeneity of study populations may underlie the conflicts in previous reports. Therefore, we performed a multicenter cohort study with 8520 unselected critically ill patients to further explore the association between serum AG and mortalities.

In our study, serum AG was adjusted by serum albumin when serum albumin was beyond the normal range, to reflect the actual AG. We found that critically ill patients with serum AG > 16 mmol/L had a higher ICU and hospital mortalities than those with 8 ≤ AG ≤ 16 mmol/L. Besides, in order to explore the influence of the heterogeneity in study population, subgroup analyses were performed. The results of subgroup analyses demonstrate that the relationship between serum AG and mortalities remain robust in patients with different age, sex, ethnicity, and APACHE IV score, in those with or without heart failure, respiratory failure, renal failure, liver diseases, sepsis, and shock, and in those without coagulopathy, trauma, hypertension, and diabetes mellitus, received or nonreceived mechanical ventilation and renal replacement therapy. However, the relationships between serum AG and mortalities have no statistical significance in ICU patients with coagulopathy, trauma, hypertension, or diabetes mellitus, which needs further investigation. Furthermore, we found the predictive accuracy of APACHE IV score for mortality, which has been widely validated in critically ill patients [25], significantly increased after combined with serum AG. Additionally, the relationship between serum AG and hospital mortality is successfully verified in an external cohort. The findings indicate that serum AG might be utilized to assist in early risk stratification for ICU patients.

Generally, AG only draws attention when acid-base disorder is present or suspected. In patients without acid-based disorder, AG is usually ignored. Previous study has demonstrated that increased AG was associated with high leukocyte count and high C-reactive protein level in 4525 healthy adults [26]. However, the relationship between AG and prognosis in critically ill patients with different acid-base statuses was scarcely studied. Therefore, in this study, we also analyzed the association of serum AG with mortalities in ICU patient with different acid-base statuses. The results show that the relationship of serum AG > 16 mmol/L with increased mortalities is not influenced by different acid-base statuses, which suggest that serum AG should be highly regarded even in those with a normal acid-base status.

The pathophysiologic mechanism underlying the association between AG with mortality is not adequately understood. Previous studies illustrated that an elevation of AG mainly resulted from metabolic acidosis, where the increased serum lactate was the common cause [2, 3, 5]. However, the correlation between serum lactate and AG was controversial in several studies [19, 20, 27, 28]. Present study showed that the correlation coefficient of serum lactate with AG is 0.401 and only 57.9% patients have elevated serum lactate (≥2 mmol/L) in patients with serum AG > 16 mmol/L. Furthermore, the present study shows that high serum AG is associated with increased mortalities, even after adjusted for serum lactate. Moreover, subgroup analyses show robust associations between AG and mortalities in patients stratified by different serum lactate levels. The results indicated that there exist potential factors, other than lactate, serving as a bridge between AG and mortality. Therefore, we further conducted a correlation matrix between AG and other serum biomarkers, such as albumin, bicarbonate, creatinine, and urea nitrogen to excavate the potential factors. However, the correlations between AG and other serum biomarkers are weak. In the future, the potential mechanism between elevated AG and increased mortality may be further explored.

Apart from serum lactate, increased AG can be caused by increased phosphate, sulfate, and other organic acids resulting from metabolic acidosis. Acidic extracellular microenvironment could stimulate inflammatory responses, including the activation of neutrophils and the complement system [29–31]. A previous study also found that a high serum AG was related with elevated levels of inflammatory biomarkers (i.e. C-reactive protein and leukocyte count) in health individuals [26]. Consistently, we find that there are more patients had high leukocyte count (>10.0 × 10^9^/L) in the group with high AG (8 ≤ AG ≤ 16 mmol/L vs. AG > 16 mmol/L: 57.0% vs. 67.4%, *P* < 0.001), which imply that hyper-inflammatory response might contribute to the increased mortality.

Impaired renal function can lead to decreased excretion of unmeasured anions, which can also contribute to the elevation of AG [32]. Thus, renal dysfunction might also be the foundation under the association of AG with mortality. In addition, increased serum AG was reported to be correlated with low cardiorespiratory fitness [33], vascular calcification [34], increased blood pressure [35], high risk of ruptured abdominal aortic aneurysm [36], cardiac arrest [37], and cardiovascular death [38]. Therefore, to sum up, elevation in serum AG may reflect a complex pathophysiological condition, and it is worth to explore the underlying mechanisms in future research.

Our study also has some limitations. Firstly, the dynamic variation of serum AG was not analyzed in the present study, because we aimed to investigate the prognostic value of serum AG upon ICU admission in order to identify high-risk patients in early stage. Secondly, due to the sample size of serum AG < 8 mmol/L was small (*n* = 42), the association of decreased serum AG with prognosis cannot be concluded. The clinical significance of decreased AG, which was usually considered as laboratory error [3], may be clarified in future studies. Finally, constricted by the retrospective design of the present study, our findings remain to be further verified in future prospective studies.

## 5. Conclusions

In aggregate, this large multicenter cohort study found that initial serum AG after ICU admission was associated with increased risk of ICU mortality and hospital mortality in critically ill patients, regardless of acid-base statuses. Our findings indicated that AG might be a useful tool for the early identification of high-risk patients, and highlighted the clinical significance of serum AG, especially in patients with normal acid-base status.

## Figures and Tables

**Table 1:** Characteristics of the study subject.

Characteristics	Total (*n* = 8520)	AG< 8 mmol/L (*n* = 42)	8 ≤ AG ≤ 16 mmol/L (*n* = 3238)	AG> 16 mmol/L (*n* = 5240)	*P* ^∗^
Age> 65 years, *n* (%)	4063 (47.7)	19 (45.2)	1446 (44.7)	2598 (49.6)	<0.001
Male, *n* (%)	4671 (54.8)	21 (50.0)	1811 (55.9)	2839 (54.2)	0.238
Caucasian, *n* (%)	6680 (78.4)	32 (76.2)	2569 (79.3)	4079 (77.8)	0.251
APACHE IV, median (IQR)	73 (55-94)	64 (45-80)	63 (47-81)	79 (61-102)	<0.001
Lactate (mmol/L), median (IQR)	2.0 (1.2-3.5)	1.5 (0.7-4.0)	1.5 (1.0-2.28)	2.3 (1.4-4.3)	<0.001
AG (mmol/L), median (IQR)	17.3 (14.5-20.5)	6.9 (6.3-7.6)	13.8 (12.3-15.0)	19.6 (17.7-22.6)	<0.001
Diseases (ICD-9 codes), *n* (%)					
Heart failure	762 (8.9)	5 (11.9)	307 (9.5)	450 (8.6)	0.299
Respiratory failure	3753 (44.0)	22 (52.4)	1487 (45.9)	2244 (42.8)	0.011
Renal failure	1347 (15.8)	6 (14.3)	287 (8.9)	1054 (20.1)	<0.001
Liver diseases	561 (6.6)	0 (0)	148 (4.6)	413 (7.9)	<0.001
Coagulopathy	395 (4.6)	2 (4.8)	120 (3.7)	273 (5.2)	0.006
Sepsis	2089 (24.5)	4 (9.5)	497 (15.3)	1588 (30.3)	<0.001
Shock	2538 (29.8)	10 (23.8)	666 (20.6)	1862 (35.5)	<0.001
Trauma	515 (6.0)	3 (7.1)	241 (7.4)	271 (5.2)	<0.001
Hypertension	791 (9.3)	5 (11.9)	388 (12.0)	398 (7.6)	<0.001
Diabetes mellitus	874 (10.3)	4 (9.5)	300 (9.3)	570 (10.9)	0.058
Treatment					
Renal replacement therapy	145 (1.7)	0 (0)	13 (0.4)	132 (2.5)	<0.001
Mechanical ventilation	3729 (43.8)	21 (50.0)	1506 (46.5)	2202 (42.0)	<0.001
Mortalities, *n* (%)					
ICU mortality	1222 (14.3)	7 (16.7)	261 (8.1)	954 (18.2)	<0.001
Hospital mortality	1809 (21.2)	8 (19.0)	406 (12.5)	1395 (26.6)	<0.001

^∗^ The difference between patients with serum AG < 8 mmol/L, 8 ≤ AG ≤ 16 mmol/L, and AG > 16 mmol/L. AG, anion gap; APACHE, acute physiology and chronic health evaluation; ICD, international classification of diseases 9th edition; ICU, intensive care unit; IQR, interquartile range.

**Table 2:** The association between serum AG and mortalities in all patients.

	AG < 8 mmol/L		8 ≤ AG ≤ 16 mmol/L		AG > 16 mmol/L	
	OR (95% CI)	*P*	OR (95% CI)	*P*	OR (95% CI)	*P*
ICU mortality						
Model 1	2.281 (1.003-5.186)	0.049	1(ref)		2.539 (2.197-2.934)	<0.001
Model 2	2.270 (0.965-5.339)	0.060	1(ref)		1.851 (1.592-2.151)	<0.001
Model 3	2.308 (0.977-5.452)	0.056	1(ref)		1.834 (1.571-2.141)	<0.001
Model 4	2.493 (1.034-6.014)	0.042	1(ref)		1.533 (1.308-1.797)	<0.001
Model 5	2.504 (1.039-6.035)	0.041	1(ref)		1.530 (1.305-1.794)	<0.001
Hospital mortality						
Model 1	1.641 (0.755-3.570)	0.211	1(ref)		2.531 (2.243-2.855)	<0.001
Model 2	1.593 (0.706-3.594)	0.262	1(ref)		1.890 (1.665-2.145)	<0.001
Model 3	1.587 (0.700-3.595)	0.269	1(ref)		1.866 (1.638-2.125)	<0.001
Model 4	1.691 (0.737-3.881)	0.215	1(ref)		1.611 (1.409-1.841)	<0.001
Model 5	1.700 (0.742-3.895)	0.210	1(ref)		1.618 (1.415-1.849)	<0.001

Model 1: unadjusted; model 2: adjusted for age, gender, ethnicity, and APACHE IV score; model 3: adjusted for age, gender, ethnicity, APACHE IV score, heart failure, respiratory failure, renal failure, liver diseases, coagulopathy, sepsis, trauma, shock, hypertension, and diabetes mellitus; model 4: adjusted for age, gender, ethnicity, APACHE IV score, heart failure, respiratory failure, renal failure, liver diseases, coagulopathy, sepsis, trauma, shock, hypertension, diabetes mellitus, and serum lactate; model 5: adjusted for age, gender, ethnicity, APACHE IV score, heart failure, respiratory failure, renal failure, liver diseases, coagulopathy, sepsis, trauma, shock, hypertension, and diabetes mellitus, serum lactate, renal replacement therapy, and mechanical ventilation. AG, anion gap; APACHE, acute physiology and chronic health evaluation; CI, confidence interval; ICU, intensive care unit; OR, odds ratio.

**Table 3:** The mortalities in patients with different acid-base disorders.

	Total	AG< 8 mmol/L	8 ≤ AG ≤ 16 mmol/L	AG> 16 mmol/L	*P* ^†^
Normal^∗^					
Subjects, *n* (%)	1167	1 (0.1)	590 (50.6)	576 (49.4)	
ICU mortality, *n* (%)	114 (9.8)	1 (100.0)	34 (5.8)	79 (13.7)	<0.001
Hospital mortality, *n* (%)	187 (16.0)	1 (100.0)	60 (10.2)	126 (21.9)	<0.001
Acid-base disorder with a normal blood pH^#^					
Subjects, *n* (%)	2432	11 (0.5)	1007 (41.4)	1414 (58.1)	
ICU mortality, *n* (%)	276 (11.3)	0 (0)	77 (7.6)	199 (14.1)	<0.001
Hospital mortality, *n* (%)	468 (19.2)	1 (9.1)	133 (13.2)	334 (23.6)	<0.001
Blood pH < 7.35					
Subjects, *n* (%)	3785	27 (0.7)	1186 (31.3)	2572 (68.0)	
ICU mortality, *n* (%)	698 (18.4)	6 (22.2)	115 (9.7)	577 (22.4)	<0.001
Hospital mortality, *n* (%)	952 (25.2)	6 (22.2)	155 (13.1)	791 (30.8)	<0.001
Blood pH>7.45					
Subjects, *n* (%)	1136	3 (0.3)	455 (40.1)	678 (59.7)	
ICU mortality, *n* (%)	134 (11.8)	0 (0)	35 (7.7)	99 (14.6)	0.001
Hospital mortality, *n* (%)	202 (17.8)	0 (0)	58 (12.7)	144 (21.2)	0.001

^∗^ Patients with 7.35 ≤ blood pH ≤ 7.45, 22 ≤ HCO_3_^−^ ≤ 26 mmol/L, and 35 ≤ P_a_CO_2_ ≤ 45 mmHg; ^#^ patients with 7.35 ≤ blood pH ≤ 7.45 and abnormal HCO_3_^−^ or P_a_CO_2_; ^†^*P* values indicated the differences of mortalities between groups with serum AG > 16 mmol/L and 8 ≤ AG ≤ 16 mmol/L. AG, anion gap; ICU, intensive care unit.

**Table 4:** The association between serum AG and mortalities in patients stratified by serum lactate.

	AG< 8 mmol/L		8 ≤ AG ≤ 16 mmol/L		AG> 16 mmol/L	
	OR (95% CI)	*P*	OR (95% CI)	*P*	OR (95% CI)	*P*
*Serum lactate < 2 mmol/L*						
ICU mortality						
Model 1	2.733 (1.039-7.186)	0.042	1(ref)		1.492 (1.193-1.867)	<0.001
Model 2	2.896 (1.062-7.895)	0.038	1(ref)		1.151 (0.912-1.451)	0.236
Model 3	3.046 (1.117-8.304)	0.029	1(ref)		1.177 (0.926-1.497)	0.182
Hospital mortality						
Model 1	1.986 (0.812-4.862)	0.133	1(ref)		1.701 (1.425-2.029)	<0.001
Model 2	2.016 (0.791-5.139)	0.142	1(ref)		1.345 (1.118-1.618)	0.002
Model 3	1.989 (0.782-5.063)	0.149	1(ref)		1.395 (1.153-1.686)	0.001
*Serum lactate ≥ 2 mmol/L*						
ICU mortality						
Model 1	1.991 (0.409-9.692)	0.394	1(ref)		2.382 (1.936-2.930)	<0.001
Model 2	1.598 (0.315-8.094)	0.571	1(ref)		1.906 (1.539-2.359)	<0.001
Model 3	1.535 (0.299-7.892)	0.608	1(ref)		1.902 (1.528-2.367)	<0.001
Hospital mortality						
Model 1	1.274 (0.263-6.187)	0.763	1(ref)		2.393 (2.000-2.863)	<0.001
Model 2	1.004 (0.198-5.087)	0.997	1(ref)		1.918 (1.591-2.312)	<0.001
Model 3	0.978 (0.190-5.042)	0.979	1(ref)		1.870 (1.544-2.265)	<0.001

Model 1: unadjusted; model 2: adjusted for age, gender, ethnicity, and APACHE IV score; model 3: adjusted for age, gender, ethnicity, APACHE IV score, heart failure, respiratory failure, renal failure, liver diseases, coagulopathy, sepsis, trauma, shock, hypertension, and diabetes mellitus. AG, anion gap; APACHE, acute physiology and chronic health evaluation; CI, confidence interval; ICU, intensive care unit; OR, odds ratio.

**Table 5:** The association between serum AG and hospital mortality in external cohort.

Models	AG < 8 mmol/L		8 ≤ AG ≤ 16 mmol/L		AG > 16 mmol/L	
	OR (95% CI)	*P*	OR (95% CI)	*P*	OR (95% CI)	*P*
Model 1	1.934 (0.797-4.694)	0.145	1(ref)		1.671 (1.097-2.546)	0.017
Model 2	1.898 (0.780-4.620)	0.158	1(ref)		1.717 (1.126-2.619)	0.012
Model 3	2.041 (0.808-5.154)	0.131	1(ref)		1.667 (1.065-2.607)	0.025

Model 1: unadjusted; model 2: adjusted for age and gender; model 3: adjusted for age, gender, heart failure, respiratory failure, renal failure, liver diseases, coagulopathy, sepsis, trauma, shock, hypertension, and diabetes mellitus; AG, anion gap; CI, confidence interval; ICU, intensive care unit; OR, odds ratio.

## Data Availability

Data are available upon reasonable request. The datasets used and/or analyzed during the current study are available from the corresponding author on reasonable request.

## Abbreviations

AG: Anion gap
APACHE: Acute physiology and chronic health evaluation
CI: Confidence interval
eICU-CRD: eICU collaborative research database
ICD-9: International classification of diseases, 9th edition
ICU: Intensive care unit
OR: Odds ratio.

## Supplementary Materials

Supplementary Materials Supplementary Table 1: the association between serum anion gap and mortalities in patients in different subgroups. Supplementary Table 2: characteristics of the study subject in external cohort. Supplementary Figure 1: the correlation matrix between AG and other serum biomarkers determined by the Spearman's correlation coefficient.
