# Supplementary material for: Relationship of Admission Serum Anion Gap and Prognosis of Critically Ill Patients: A Large Multicenter Cohort Study
Source: Dis Markers. 2022 Dec 14;2022:5926049. doi: 10.1155/2022/5926049 (PMC9771639; doi:10.1155/2022/5926049)
Supplement: Supplementary Materials — Supplementary Table 1: the association between serum anion gap and mortalities in patients in different subgroups. Supplementary Table 2: characteristics of the study subject in external cohort. Supplementary Figure 1: the correlation matrix between AG and other serum biomarkers determined by the Spearman's correlation coefficient. [file 5926049.f1.docx]

**Supplemental Digital Content**

**Supplementary Table 1** The association between serum anion gap and mortalities in patients in different subgroups

**Supplementary Table 2** Characteristics of the study subject in external cohort

**Supplementary Figure 1** The correlation matrix between AG and other serum biomarkers determined by the Spearman’s correlation coefficient

**Supplementary Table 1** The association between serum anion gap and mortalities in patients in different subgroups

|  | **ICU mortality** | | **Hospital mortality** | |
| --- | --- | --- | --- | --- |
|  | OR (95% CI) | *P* | OR (95% CI) | *P* |
| **Age** |  |  |  |  |
| **≤65 years** |  |  |  |  |
| AG<8 mmol/L | 4.036 (1.224-13.302) | 0.022 | 2.777 (0.860-8.973) | 0.088 |
| 8≤AG≤16 mmol/L | 1 (ref) |  | 1 (ref) |  |
| AG>16 mmol/L | 1.629 (1.284-2.066) | <0.001 | 1.841 (1.492-2.272) | <0.001 |
| **>65 years** |  |  |  |  |
| AG<8 mmol/L | 1.572 (0.422-5.858) | 0.500 | 1.125 (0.352-3.597) | 0.843 |
| 8≤AG≤16 mmol/L | 1 (ref) |  | 1 (ref) |  |
| AG>16 mmol/L | 1.454 (1.174-1.801) | 0.001 | 1.463 (1.23-1.742) | <0.001 |
| **Sex** |  |  |  |  |
| **Male** |  |  |  |  |
| AG<8 mmol/L | 4.131 (1.367-12.483) | 0.012 | 3.640 (1.298-10.208) | 0.014 |
| 8≤AG≤16 mmol/L | 1 (ref) |  | 1 (ref) |  |
| AG>16 mmol/L | 1.474 (1.194-1.821) | <0.001 | 1.686 (1.407-2.021) | <0.001 |
| **Femal** |  |  |  |  |
| AG<8 mmol/L | 1.149 (0.247-5.335) | 0.859 | 0.578 (0.126-2.647) | 0.480 |
| 8≤AG≤16 mmol/L | 1 (ref) |  | 1 (ref) |  |
| AG>16 mmol/L | 1.631 (1.279-2.079) | <0.001 | 1.527 (1.252-1.863) | <0.001 |
| **Ethnicity** |  |  |  |  |
| **Caucasian** |  |  |  |  |
| AG<8 mmol/L | 1.749 (0.571-5.357) | 0.328 | 1.355 (0.488-3.760) | 0.559 |
| 8≤AG≤16 mmol/L | 1 (ref) |  | 1 (ref) |  |
| AG>16 mmol/L | 1.467 (1.231-1.748) | <0.001 | 1.564 (1.347-1.815) | <0.001 |
| **Other** |  |  |  |  |
| AG<8 mmol/L | 6.590 (1.437-30.218) | 0.015 | 2.879 (0.655-12.647) | 0.161 |
| 8≤AG≤16 mmol/L | 1 (ref) |  | 1 (ref) |  |
| AG>16 mmol/L | 1.914 (1.300-2.82) | 0.001 | 1.813 (1.338-2.457) | <0.001 |
| **APACHE IV** |  |  |  |  |
| **≤73** |  |  |  |  |
| AG<8 mmol/L | 2.230 (0.503-9.879) | 0.291 | 2.076 (0.602-7.163) | 0.248 |
| 8≤AG≤16 mmol/L | 1 (ref) |  | 1 (ref) |  |
| AG>16 mmol/L | 1.468 (1.103-1.954) | 0.009 | 1.677 (1.342-2.096) | <0.001 |
| **>73** |  |  |  |  |
| AG<8 mmol/L | 2.651 (0.872-8.058) | 0.086 | 1.490 (0.497-4.469) | 0.476 |
| 8≤AG≤16 mmol/L | 1 (ref) |  | 1 (ref) |  |
| AG>16 mmol/L | 1.556 (1.285-1.884) | <0.001 | 1.572 (1.33-1.858) | <0.001 |
| **Heart failure** |  |  |  |  |
| **Yes** |  |  |  |  |
| AG<8 mmol/L | 3.579 (0.352-36.430) | 0.281 | 2.017 (0.209-19.511) | 0.544 |
| 8≤AG≤16 mmol/L | 1 (ref) |  | 1 (ref) |  |
| AG>16 mmol/L | 2.063 (1.22-3.497) | 0.007 | 2.401 (1.563-3.688) | <0.001 |
| **No** |  |  |  |  |
| AG<8 mmol/L | 2.329 (0.895-6.059) | 0.083 | 1.660 (0.677-4.070) | 0.268 |
| 8≤AG≤16 mmol/L | 1 (ref) |  | 1 (ref) |  |
| AG>16 mmol/L | 1.479 (1.252-1.747) | <0.001 | 1.536 (1.334-1.769) | <0.001 |
| **Respiratory failure** |  |  |  |  |
| **Yes** |  |  |  |  |
| AG<8 mmol/L | 3.669 (1.316-10.227) | 0.013 | 2.659 (0.999-7.080) | 0.050 |
| 8≤AG≤16 mmol/L | 1 (ref) |  | 1 (ref) |  |
| AG>16 mmol/L | 1.473 (1.195-1.817) | <0.001 | 1.52 (1.268-1.822) | <0.001 |
| **No** |  |  |  |  |
| AG<8 mmol/L | 0.938 (0.119-7.368) | 0.952 | 0.502 (0.064-3.914) | 0.511 |
| 8≤AG≤16 mmol/L | 1 (ref) |  | 1 (ref) |  |
| AG>16 mmol/L | 1.629 (1.274-2.082) | <0.001 | 1.738 (1.424-2.123) | <0.001 |
| **Renal failure** |  |  |  |  |
| **Yes** |  |  |  |  |
| AG<8 mmol/L | 1.238 (0.128-12.021) | 0.854 | 0.722 (0.077-6.779) | 0.776 |
| 8≤AG≤16 mmol/L | 1 (ref) |  | 1 (ref) |  |
| AG>16 mmol/L | 1.333 (0.863-2.061) | 0.195 | 1.534 (1.056-2.228) | 0.025 |
| **No** |  |  |  |  |
| AG<8 mmol/L | 2.850 (1.103-7.364) | 0.031 | 2.004 (0.824-4.873) | 0.125 |
| 8≤AG≤16 mmol/L | 1 (ref) |  | 1 (ref) |  |
| AG>16 mmol/L | 1.558 (1.313-1.847) | <0.001 | 1.616 (1.4-1.865) | <0.001 |
| **Liver diseases** |  |  |  |  |
| **Yes** |  |  |  |  |
| AG<8 mmol/L | /^*^ | /^*^ | /^*^ | /^*^ |
| 8≤AG≤16 mmol/L | 1 (ref) |  | 1 (ref) |  |
| AG>16 mmol/L | 1.959 (1.039-3.693) | 0.038 | 2.601 (1.466-4.612) | 0.001 |
| **No** |  |  |  |  |
| AG<8 mmol/L | 2.446 (1.015-5.895) | 0.046 | 1.653 (0.721-3.790) | 0.235 |
| 8≤AG≤16 mmol/L | 1 (ref) |  | 1 (ref) |  |
| AG>16 mmol/L | 1.513 (1.284-1.784) | <0.001 | 1.572 (1.37-1.804) | <0.001 |
| **Coagulopathy** |  |  |  |  |
| **Yes** |  |  |  |  |
| AG<8 mmol/L | /^*^ | /^*^ | /^*^ | /^*^ |
| 8≤AG≤16 mmol/L | 1 (ref) |  | 1 (ref) |  |
| AG>16 mmol/L | 1.081 (0.568-2.057) | 0.812 | 1.387 (0.774-2.485) | 0.271 |
| **No** |  |  |  |  |
| AG<8 mmol/L | 2.848 (1.169-6.937) | 0.021 | 1.894 (0.818-4.384) | 0.136 |
| 8≤AG≤16 mmol/L | 1 (ref) |  | 1 (ref) |  |
| AG>16 mmol/L | 1.576 (1.336-1.858) | <0.001 | 1.625 (1.416-1.865) | <0.001 |
| **Sepsis** |  |  |  |  |
| **Yes** |  |  |  |  |
| AG<8 mmol/L | /^*^ | /^*^ | /^*^ | /^*^ |
| 8≤AG≤16 mmol/L | 1 (ref) |  | 1 (ref) |  |
| AG>16 mmol/L | 1.509 (1.099-2.074) | 0.011 | 1.45 (1.1-1.909) | 0.008 |
| **No** |  |  |  |  |
| AG<8 mmol/L | 3.188 (1.295-7.849) | 0.012 | 2.135 (0.919-4.961) | 0.078 |
| 8≤AG≤16 mmol/L | 1 (ref) |  | 1 (ref) |  |
| AG>16 mmol/L | 1.543 (1.284-1.855) | <0.001 | 1.673 (1.435-1.95) | <0.001 |
| **Shock** |  |  |  |  |
| **Yes** |  |  |  |  |
| AG<8 mmol/L | 5.040 (1.218-20.860) | 0.026 | 3.217 (0.766-13.522) | 0.111 |
| 8≤AG≤16 mmol/L | 1 (ref) |  | 1 (ref) |  |
| AG>16 mmol/L | 1.511 (1.157-1.975) | 0.002 | 1.449 (1.144-1.8334) | 0.002 |
| **No** |  |  |  |  |
| AG<8 mmol/L | 1.641 (0.469-5.737) | 0.438 | 1.272 (0.424-3.818) | 0.668 |
| 8≤AG≤16 mmol/L | 1 (ref) |  | 1 (ref) |  |
| AG>16 mmol/L | 1.566 (1.285-1.909) | <0.001 | 1.709 (1.452-2.01) | <0.001 |
| **Trauma** |  |  |  |  |
| **Yes** |  |  |  |  |
| AG<8 mmol/L | 8.419 (0.627-113.130) | 0.108 | 3.365 (0.269-42.033) | 0.346 |
| 8≤AG≤16 mmol/L | 1 (ref) |  | 1 (ref) |  |
| AG>16 mmol/L | 1.435 (0.779-2.642) | 0.247 | 1.269 (0.755-2.132) | 0.368 |
| **No** |  |  |  |  |
| AG<8 mmol/L | 2.294 (0.893-5.895) | 0.085 | 1.625 (0.672-3.930) | 0.281 |
| 8≤AG≤16 mmol/L | 1 (ref) |  | 1 (ref) |  |
| AG>16 mmol/L | 1.553 (1.317-1.833) | <0.001 | 1.654 (1.439-1.9) | <0.001 |
| **Hypertension** |  |  |  |  |
| **Yes** |  |  |  |  |
| AG<8 mmol/L | 7.501 (0.885-63.562) | 0.065 | 3.476 (0.459-26.344) | 0.228 |
| 8≤AG≤16 mmol/L | 1 (ref) |  | 1 (ref) |  |
| AG>16 mmol/L | 0.715 (0.414-1.234) | 0.228 | 0.818 (0.525-1.274) | 0.374 |
| **No** |  |  |  |  |
| AG<8 mmol/L | 2.041 (0.741-5.625) | 0.168 | 1.428 (0.559-3.653) | 0.457 |
| 8≤AG≤16 mmol/L | 1 (ref) |  | 1 (ref) |  |
| AG>16 mmol/L | 1.658 (1.402-1.963) | <0.001 | 1.735 (1.506-1.999) | <0.001 |
| **Diabetes mellitus** |  |  |  |  |
| **Yes** |  |  |  |  |
| AG<8 mmol/L | 4.277 (0.383-47.779) | 0.238 | 1.378 (0.131-14.496) | 0.789 |
| 8≤AG≤16 mmol/L | 1 (ref) |  | 1 (ref) |  |
| AG>16 mmol/L | 1.034 (0.612-1.747) | 0.902 | 1.005 (0.662-1.526) | 0.980 |
| **No** |  |  |  |  |
| AG<8 mmol/L | 2.451 (0.948-6.335) | 0.064 | 1.784 (0.731-4.352) | 0.203 |
| 8≤AG≤16 mmol/L | 1 (ref) |  | 1 (ref) |  |
| AG>16 mmol/L | 1.601 (1.355-1.893) | <0.001 | 1.698 (1.473-1.956) | <0.001 |
| **Renal replacement therapy** |  |  |  |  |
| **Yes** |  |  |  |  |
| AG<8 mmol/L | /^*^ | /^*^ | /^*^ | /^*^ |
| 8≤AG≤16 mmol/L | /^*^ | /^*^ | /^*^ | /^*^ |
| AG>16 mmol/L | /^*^ | /^*^ | /^*^ | /^*^ |
| **No** |  |  |  |  |
| AG<8 mmol/L | 2.496 (1.035-6.019) | 0.042 | 1.686 (0.734-3.871) | 0.218 |
| 8≤AG≤16 mmol/L | 1 (ref) |  | 1 (ref) |  |
| AG>16 mmol/L | 1.537 (1.310-1.804) | <0.001 | 1.614 (1.411-1.846) | <0.001 |
| **Mechanical ventilation** |  |  |  |  |
| **Yes** |  |  |  |  |
| AG<8 mmol/L | 6.449 (2.151-19.332) | 0.001 | 4.310 (1.547-12.007) | 0.005 |
| 8≤AG≤16 mmol/L | 1 (ref) |  | 1 (ref) |  |
| AG>16 mmol/L | 1.830 (1.448-2.312) | <0.001 | 1.781 (1.468-2.161) | <0.001 |
| **No** |  |  |  |  |
| AG<8 mmol/L | 0.829 (0.179-3.842) | 0.810 | 0.496 (0.109-2.264) | 0.366 |
| 8≤AG≤16 mmol/L | 1 (ref) |  | 1 (ref) |  |
| AG>16 mmol/L | 1.324 (1.065-1.644) | 0.011 | 1.473 (1.224-1.772) | <0.001 |

^*^ The number of patients is too small to assess the differences between groups. Multivariable logistic regression models were adjusted for age, gender, ethnicity, APACHE IV score, heart failure, respiratory failure, renal failure, liver diseases, coagulopathy, sepsis, trauma, shock, hypertension, and diabetes mellitus, and serum lactate. AG, anion gap; APACHE, Acute Physiology and Chronic Health Evaluation; CI, confidence interval; ICU, intensive care unit; OR, odds ratio.

**Supplementary Table 2** Characteristics of the study subject in external cohort

| **Characteristics** | **Total**  **(n=1873)** | **AG<8 mmol/L**  **(n=79)** | **8≤AG≤16** **mmol/L**  **(n=1055)** | **AG>16** **mmol/L**  **(n=739)** | ***P^*^*** |
| --- | --- | --- | --- | --- | --- |
| Age >65 years, n (%) | 1114 (59.5) | 50 (4.5) | 610 (57.8) | 454 (61.4) | 0.240 |
| Male, n (%) | 1138 (60.8) | 58 (73.4) | 655 (62.1) | 425 (57.5) | 0.009 |
| AG (mmol/L), median (IQR) | 14.6 (11.7-18.3) | 6.5 (5.5-7.4) | 12.5 (10.9-14.2) | 19.5 (17.5-23.3) | <0.001 |
| Diseases (ICD-10 codes), n (%) |  |  |  |  |  |
| Heart failure | 423 (22.6) | 18 (22.8) | 190 (18.0) | 215 (29.1) | <0.001 |
| Respiratory failure | 774 (41.3) | 48 (60.8) | 423 (40.1) | 303 (41.0) | 0.002 |
| Renal failure | 94 (5.0) | 2 (2.5) | 44 (4.2) | 48 (6.5) | 0.050 |
| Liver diseases | 541 (28.9) | 16 (20.3) | 277 (26.3) | 248 (33.6) | <0.001 |
| Coagulopathy | 307 (16.4) | 8 (10.1) | 125 (11.8) | 174 (23.5) | <0.001 |
| Sepsis | 8 (0.4) | 0 (0) | 5 (0.5) | 3 (0.4) | 0.818 |
| Shock | 693 (37.0) | 24 (30.4) | 452 (42.8) | 217 (29.4) | <0.001 |
| Trauma | 579 (30.9) | 20 (25.3) | 343 (32.5) | 216 (29.2) | 0.182 |
| Hypertension | 751 (40.1) | 26 (32.9) | 432 (40.9) | 293 (39.6) | 0.354 |
| Diabetes mellitus | 347 (18.5) | 4 (5.1) | 148 (14.0) | 195 (26.4) | <0.001 |
| Hospital mortality | 98 (5.2) | 6 (7.6) | 43 (4.1) | 49 (6.6) | 0.036 |

* The difference between patients with serum AG<8 mmol/L, 8≤AG≤16 mmol/L, and AG>16 mmol/L. AG, anion gap; ICD, International Classification of Diseases 9th Edition; ICU, intensive care unit; IQR, inter-quartile range.


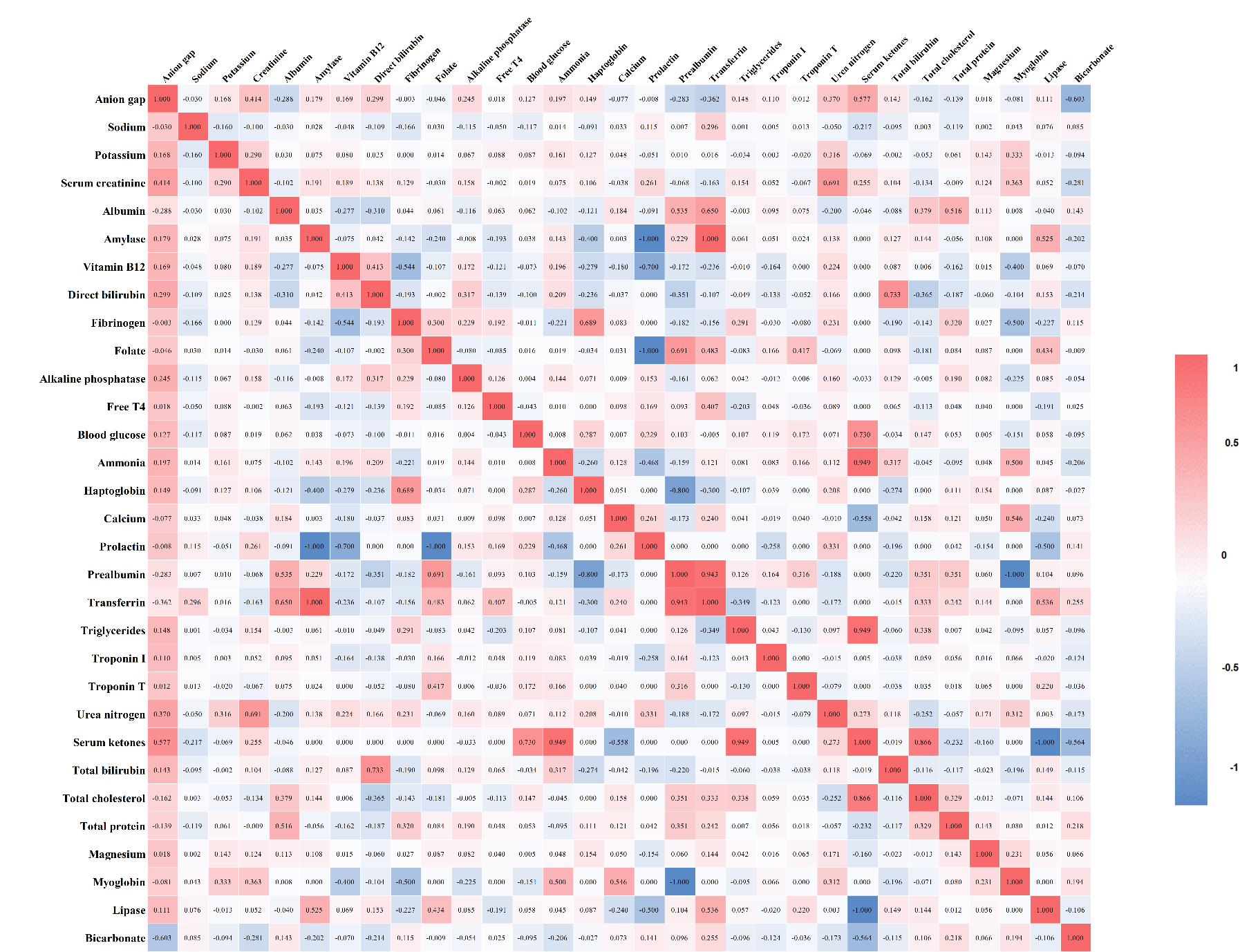


**Supplementary Figure 1** The correlation matrix between AG and other serum biomarkers determined by the Spearman’s correlation coefficient
